# Enhancing patient involvement through shared decision-making: Are we there yet in multiple myeloma? Findings from a cross-country survey

**DOI:** 10.1371/journal.pone.0356989

**Published:** 2026-08-28

**Authors:** Charlotte Verbeke, Jolien Broekmans, Elise Schoefs, Silène ten Seldam, Kate Morgan, Katie Joyner, Eilidh Duncan, Ariel Aviv, Varda Shoham, Michel Delforge, Anneleen Vanhellemont, Rosanne Janssens, Alexandre Bohyn, Isabelle Huys

**Affiliations:** 1 Department of Pharmaceutical and Pharmacological Sciences, KU Leuven, Leuven, Belgium; 2 Myeloma Patients Europe, Brussels, Belgium; 3 Department of Hematology, Ha’Emek medical center, Afula, Israel; 4 AMEN association, Kiron, Israel; 5 Department of Hematology, University Hospital Leuven, Leuven, Belgium; 6 Department of Public Health and Primary Care, Leuven Biostatistics and Statistical Bioinformatics Centre, KU Leuven, Leuven, Belgium; Tekirdag Namik Kemal University: Tekirdag Namik Kemal Universitesi, TÜRKIYE

## Abstract

**Introduction:**

Shared decision-making (SDM) can enhance patient involvement in treatment and care decisions. Still, questions remain on how multiple myeloma (MM) patients perceive and evaluate their current and desired roles in these decisions and whether characteristics influence their willingness to be involved. This study aimed to identify I) the current knowledge of the concept SDM among MM patients and healthcare professionals (HCPs) involved in MM care, II) how MM patients are and want to be involved in decisions, and III) differences in the willingness to be involved in view of participants’ characteristics (e.g., age, gender, health literacy).

**Methods:**

An online survey for MM patients and HCPs involved in MM care was launched in October and December 2023, respectively. The data was statistically analyzed.

**Results:**

558 patients and 89 HCPs completed the survey. Most patients lived in the Netherlands (28.7%), Israel (23.5%) and Belgium (15.8%), and most HCPs lived in France (39.3%) and Israel (24.7%). 56.6% of the patients and 28.1% of the HCPs had not previously heard of SDM. Almost all patients (96.1%) indicated wanting to be involved in treatment decisions. 3.9% of patients indicated not wanting to be involved, because they did not know whether they would have enough knowledge to decide or because they were used to the HCP deciding. All HCPs believed that MM patients should be involved if they want to. Patients had higher involvement scores at recent consultations and desired to be more involved in initial decisions following diagnosis. The survey revealed no significant differences in participants’ involvement and their characteristics.

**Conclusion:**

MM patients want to be involved in decisions and HCPs are willing to involve MM patients. However, challenges remain concerning the practical implementation of involvement and SDM. This highlights the importance of understanding SDM and patient involvement and assessing the individual needs and preferences of MM patients.

## Introduction

Involving patients in their treatment and care is considered a fundamental goal of modern healthcare, as it has been associated with several benefits, including improved satisfaction and therapy adherence, as well as greater patient empowerment and a strong sense of control [1,2].

Shared decision-making (SDM) can support and facilitate patient involvement in treatment and care decisions. Within the SDM process, patients and treating physicians make treatment decisions together, ensuring that choices reflect both patient preferences (e.g., regarding mode and location of administration, side effects management, and work and family priorities) and clinicians’ expertise [3].

Several models have conceptualized SDM and the roles of those involved [4–7]. One widely recognized is the three talk model of Elwyn et al. [7], which describes SDM between patients and healthcare professionals (HCPs) as a three-step process: I) ‘team talk’ in which the clinician emphasizes that a decision needs to be made and that patient and clinician work together as a team, while offering support and exploring the patient’s goals and preferred level of involvement, II) ‘option talk’, in which available treatment and care options are discussed, and III) ‘decision talk’, in which a treatment decision is made based on patients-informed preferences.

Applying SDM is particularly important in preference-sensitive contexts (i.e., a context where multiple treatment options exist, where evidence supporting one option is uncertain or variable, or where patients’ views about the most important benefits and risks differ [8]), such as oncology and more in particular multiple myeloma (MM) [8–11].

MM is a rare hematological cancer that is currently considered incurable. Many treatments exist, and the treatment landscape keeps evolving with upcoming new treatments (e.g., CAR-T cell therapy and bispecific antibodies) and treatment combinations, each with their own characteristics (e.g., effects on survival, efficacy, toxicity) [12–15].

Guidelines (e.g., ASCO and ESMO guidelines) list treatment options that HCPs and patients can further discuss [13,15]. In the ASCO guidelines the necessity of SDM, given the many effective treatment options available for both newly diagnosed and relapsed and refractory MM patients, and the importance of active participation of patients in decision-making, is highlighted [13]. In some countries where treatment options are limited [16], SDM can still be of added value in psychosocial care or other care aspects [17,18].

Some research has been conducted among MM patients and/or HCPs involved in the treatment of MM, investigating elements of SDM or patient involvement. One interview study focused on the development of trust between MM patients and clinicians, showing that communication training and SDM tools can enhance this trust development [19].

Other interview studies aimed to get insights into the perspectives of HCPs [20] and MM patients [21,22], regarding SDM, revealing that patients want to be involved in different ways and to different extents [22]. The studies also identified challenges, barriers and facilitators for the implementation of SDM in MM care [20,21]. A survey study among relapsed/refractory MM patients and HCPs in the USA investigated their perspectives on the treatment process, more particular the extent and nature of SDM and how patients and physicians communicate during treatment selection [23]. Although some research has been performed showing important elements and the relevance of SDM and involving patients in MM care, questions remain regarding how MM patients perceive and evaluate both their current and desired involvement and their roles in treatment and care decision-making. Moreover, patients’ characteristics that may influence willingness to participate in these decisions seem underexplored.

To investigate these gaps, a project was set up together with the patient organization *Myeloma Patients Europe* (MPE), consisting of different steps including our prior interview study with MM patients and HCPs in diverse countries in Europe and Israel. Findings of the study showed willingness towards SDM in MM care. However, patients reported a lack of assessment of their desired involvement and their preferences and needs. The interviews also revealed that the preferred level of involvement in the SDM process seemed to be highly individual among MM patients [21].

Following the interview study, this survey study was conducted aiming to quantify elements of patients’ involvement and SDM and to provide insights on I) the current knowledge of the concept SDM among MM patients and HCPs involved in MM care, II) current and desired involvement of MM patients in clinical decision-making, and III) possible differences in the willingness to be involved related to participants’ characteristics.

## Methods

### Survey development

An English survey was created for MM patients and HCPs involved in MM care. The surveys for both groups were largely similar, with specific questions adapted where needed for each target group. The patient organization MPE and a steering committee (consisting of MM patients (n = 3), researchers (n = 2), haematologists (n = 3), and a caregiver (n = 1)) provided feedback on the survey. The survey was translated into different languages (Dutch, French, Spanish, German, Swedish, Slovenian, Hebrew) to give MM patients and HCPs the opportunity to answer in their preferred language and was reviewed by native speaking patients and HCPs. The questions were implemented in Qualtrics software. To ensure clarity and understandability, the survey was piloted with MM patients (n = 3) and HCPs involved in MM (n = 3) and further refined based on the received feedback.

The survey consisted of different parts. An introduction and background information section were provided followed by the survey explanation and questions. The survey questions were created around four main themes: I) participant characteristics, II) patient involvement, III) sources of information, and IV) roles in decision-making (S1 Appendix). In this paper the main focus will be on the questions related to patient involvement.

The survey included a combination of closed-ended multiple choice questions and validated instruments next to survey-specific questions. For the patient survey, the validated questions included: the SDM-Q-9 [24], Chew’s Health Literacy Screening questions [25] and the visual analogue scale [26] while the HCP survey included the SDM-Q-Doc [27]. These SDM questionnaires (SDM-Q-9 and SDM-Q-Doc) consist of nine statements reflecting on different steps of the SDM process. Both questionnaires use the same scale and can be compared. The SDM-Q-9 and SDM-Q-Doc are transformed to a 0–100 score, with a higher score indicating that the SDM steps were applied to a higher extent. The Chew’s Health Literacy Screening questions aim to assess the health literacy of patients, with a higher score indicating a lower healthy literacy [25]. The visual analogue scale in the patient survey, measured the self-rated health: *“How would you currently rate your health on a scale from 0 to 100?”,* where 0 represented “poor health” and 100 represented “excellent health”.

### Participants and recruitment

The MM patient survey was distributed online from October 2023 to February 2024. This survey targeted patients over 18 years old, with a self-reported MM diagnosis. The HCP survey was distributed online from December 2023 to March 2024, targeting HCPs involved in MM care (e.g., hematologist, oncologist, hemato-oncologist, nurse specialist, nurse). The link to the online surveys was disseminated via the network of the patient organization MPE (e.g., website and social media).

Ethical approval was obtained from the Ethical Committee Research UZ/KU Leuven in Belgium (S66580). The data collection in the survey was anonymous. As advised by our Ethical Committee, participants were informed via an information letter, and no informed consent was requested.

### Survey analysis

The data of the anonymous survey was processed, and all answers were summarized as group characteristics. Participants’ characteristics were summarized in SPPS using descriptive statistics. Continuous variables were summarized using mean and standard deviation, or median and interquartile range, as appropriate. Categorical data were summarized using count and percentages. The data from the open survey questions was analyzed thematically using the framework analysis [28].

Associations between participants’ responses and several relevant characteristics were investigated. Furthermore, associations between patients’ and HCPs’ responses regarding involvement and receiving information were also investigated.

Associations between two continuous variables were tested using the Pearson correlation when the relationship was linear, and the Spearman correlation otherwise. Associations between two categorical variables were tested using the Chi Square Test, or Fisher’s exact test when some cells had a count lower than five. Finally, associations between a continuous variable and a categorical variable were tested using a t-test when the continuous variable was normally distributed, and Wilcoxon rank sum test otherwise. If the categorical variable had more than two categories, a one-way ANOVA was used to compare the group means when the assumptions of normality and homoscedasticity were met, and the non-parametric Kruskal-Wallis test was used otherwise. All tests were two-sided and performed at a significance level of 0.05. Since the study is exploratory, no correction for multiple testing was applied. All p-values should then be interpreted cautiously and be considered hypothesis-generating. All analyses were conducted using R [29].

## Results

### Participants’ characteristics

558 MM patients and 89 HCPs fully answered the survey. Patients had a mean age of 65.39 years (SD = 9.31) and included 269 females (48.2%) and 268 males (51.3%). The largest group of patients came from The Netherlands (n = 160, 28.7%), Israel (n = 131, 23.5%) and Belgium (n = 88, 15.8%). The majority reported that they did not have a medical background (e.g., doctor, nurse, pharmacist, health care personnel) (n = 472, 84.6%) and that their native language was spoken at the hospital (n = 537, 96.2%).139 of the patients (24.9%) had more than three treatment lines.

HCPs had a mean age of 47.17 years (SD = 11.15) and included 56 females (62.9%) and 31 males (34.8%). The largest group of HCPs came from France (n = 35, 39.3%) and Israel (n = 22, 24.7%). The majority of the HCPs (n = 49, 55.1%) were haematologists (Table 1 and Table 2).

**Table 1:** Patient characteristics (n = 558).

Personal characteristics	n = 558
**Age in years, Mean (SD)**	65.39 (9.31)
Gender, n (%) *	
*Female*	269 (48.21)
*Male*	286 (51.25)
*Non-binary/gender non-conforming*	1 (0.18)
*Other*	1 (0.18)
*Prefer not to say*	1 (0.18)
**Country, n (%)**	
*The Netherlands*	160 (28.7)
*Israel*	131 (23.5)
*Belgium*	88 (15.8)
*Germany*	49 (8.8)
*Sweden*	49 (8.8)
*Slovenia*	28 (5.0)
*Spain*	12 (2.2)
*Portugal*	9 (1.6)
*Switzerland*	9 (1.6)
*Finland*	3 (0.5)
*France*	3 (0.5)
*Ireland*	2 (0.4)
*Brazil*	1 (0.2)
*Czech Republic*	1 (0.2)
*Hungary*	1 (0.2)
*Italy*	1 (0.2)
*Macedonia*	1 (0.2)
*Norway*	1 (0.2)
*Romania*	1 (0.2)
**Native language spoken at the hospital, n (%)**	
*Yes*	537 (96.2)
*No*	21 (3.8)
**Highest level of education, n (%)**	
*Did not finish high school*	16 (2.9)
*High school*	160 (28.7)
*Bachelor’s degree*	155 (27.8)
*Master’s degree*	118 (21.1)
*Doctorate*	35 (6.3)
*Other*	74 (13.3)
**Medical background, n (%)**	
*Yes*	76 (13.6)
*No*	472 (84.6)
*I am not sure*	10 (1.8)
Current employment status, n (%) **	
*Student*	2 (0.3)
*Full-time*	72 (12.3)
*Part-time*	47 (8.0)
*(Currently) not/partly working due to my disease*	75 (12.8)
*Unemployed*	19 (3.2)
*Self-employed*	45 (7.7)
*Retired*	318 (54.3)
*Prefer not to say*	5 (0.9)
*Other*	3 (0.5)
With whom to go to the consultations, n (%) **	
*Alone*	257 (37.1)
*A partner*	321 (46.3)
*A child*	43 (6.2)
*A family member*	48 (6.9)
*A friend*	19 (2.7)
*Other*	5 (0.7)
**Health literacy, Mean (SD)**	2.68 (2.196) ***
**Disease and treatment related characteristics**	**n = 558**
**Health status - Visual analogue scale (VAS), Mean (SD)**	69.67 (17.96)
**Age diagnosis MM, Mean (SD)**	59.56 (9.36)
**Time since diagnosis in years Mean (SD)**	5.82 (4.92)
Treatment lines, n (%) ****	
*1 line*	106 (19.0)
*2 lines*	162 (29.0)
*3 lines*	108 (19.4)
*4 lines*	60 (10.8)
*5 lines*	40 (7.2)
*More lines*	39 (7.0)
*I am not sure*	43 (7.7)
**Treatment location, n (%)**	
*University hospital/academic centre*	181 (32.4)
*General hospital*	332 (59.5)
*I am not sure*	3 (0.5)
*Other*	42 (7.5)

*Since there are very few events for gender other than ‘male’ and ‘female’ only these two categories are kept for analysis

**Multiple options could be indicated for these questions; the percentages will not add up to 100%

***A higher score indicates a lower healthy literacy (score can ranged from 0–12) [25]

****A note was added in the survey explaining what was meant with treatment line*: A treatment ‘line’ refers to the treatment or combination of treatments prescribed to you until your disease is under control (you reach remission or stable phase). That line of treatment may include a combination of 2 or 3 anti-myeloma medicines given at the same time. A stem cell transplant is considered a separate treatment line.*

**Table 2:** HCPs characteristics (n = 89).

Personal characteristics	n = 89
**Age in years, Mean (SD)**	47.17 (11.15)
Gender, n (%) *	
*Female*	56 (62.9)
*Male*	31 (34.8)
*Non-binary/gender-non conforming*	1 (1.1)
*Other*	1 (1.1)
**Country, n (%)**	
*France*	35 (39.3)
*Israel*	22 (24.7)
*Ireland*	6 (6.7)
*Slovenia*	6 (6.7)
*Italy*	4 (4.5)
*Spain*	4 (4.5)
*United Kingdom*	3 (3.4)
*Finland*	2 (2.2)
*Belgium*	1 (1.1)
*Chili*	1 (1.1)
*Denmark*	1 (1.1)
*Ecuador*	1 (1.1)
*Germany*	1 (1.1)
*North Macedonia*	1 (1.1)
*Sweden*	1 (1.1)
**Treatment and care related characteristics**	**n = 89**
**HCP type, n (%)**	
*Hematologist*	49 (55.1)
*Hemato-oncologist*	13 (14.6)
*Nurse specialist*	9 (10.1)
*Nurse*	12 (13.5)
*Other HCP*	6 (6.7)
**Workplace, n (%)**	
*University hospital/academic centre*	39 (43.8)
*General hospital*	45 (50.6)
*Other*	5 (5.6)
**Work experience in years, Mean (SD)**	17.03 (10.22)
**Average consultation in minutes, Mean (SD)**	29.78 (16.21)
**Involvement in research, n (%)**	
*Yes*	66 (74.2)
*No*	23 (25.8)
**Involvement in development of clinical guidelines, n (%)**	
*Yes*	32 (36.0)
*No*	57 (64.0)

*Since there are very few events for gender other than ‘male’ and ‘female’ only these two categories are kept for analysis

### Knowledge of the SDM concept

**Fig 1:**
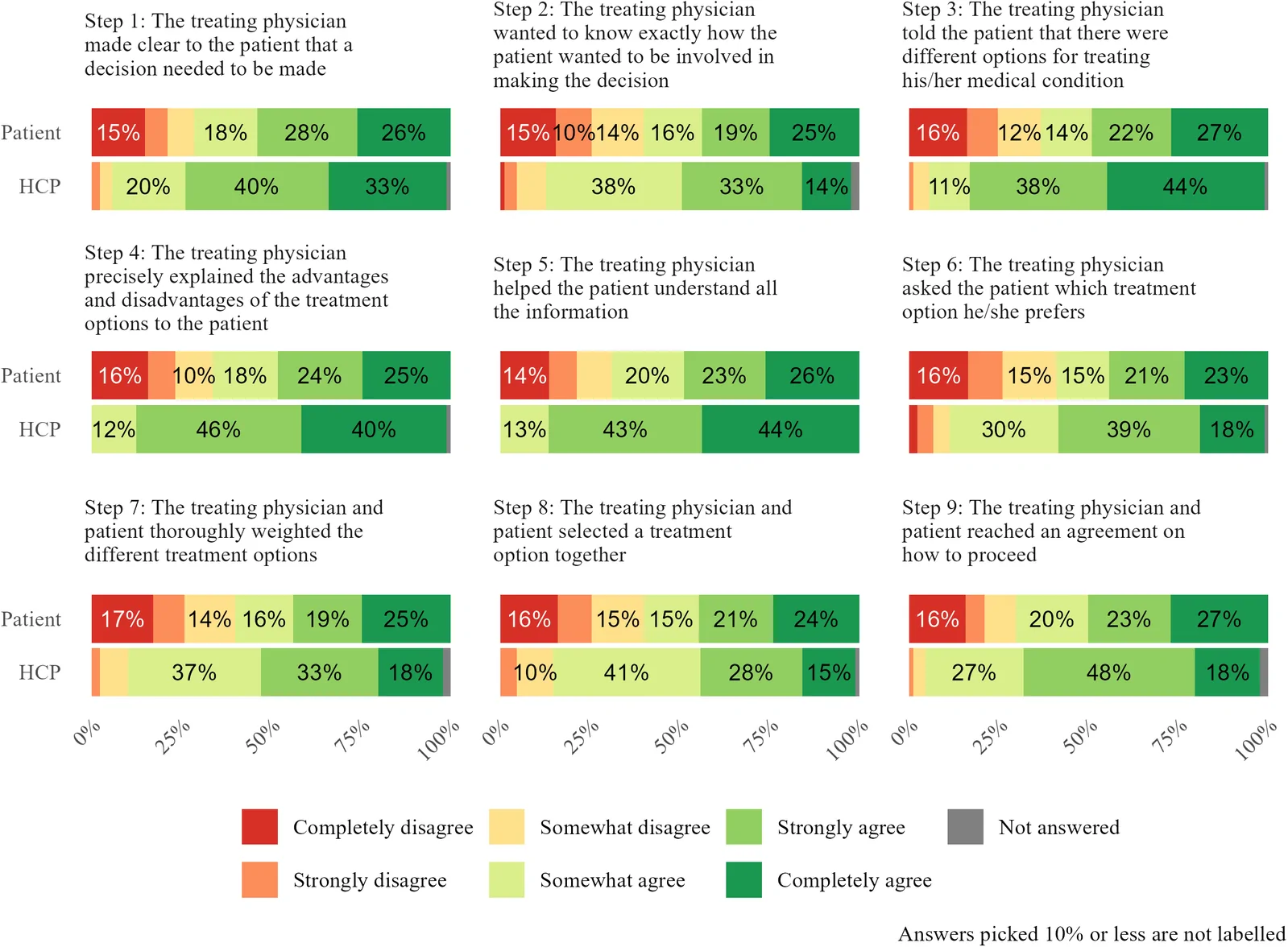
Visualization of the SDM-Q-9 and SDM-Q-Doc results. HCP: Healthcare professional. Patients were asked to reflect on a recent consultation with their treating physician where a decision had to be made and to what extent they agree with the statements (SDM-Q-9). HCPs were asked to reflect on a recent consultation where a decision had to be made for a multiple myeloma patient and to what extent they agree with the statements (SDM-Q-Doc). Both treating physicians and other members of the multidisciplinary team (e.g., nurse specialists) could participate, therefore, the answer option N/A was added in the SDM-Q-Doc questionnaire.

A total of 316 patients (56.6%) and 25 HCPs (28.1%) had not previously heard of the SDM concept. Patients (n = 242, 43.4%) and HCPs (n = 64, 71.9%) that had previously heard of the concept, were asked to describe SDM in their own words. Most common terminology by both patients and HCPs were related to ‘providing and receiving information’ (patients: n = 89, 36.8%; HCPs: n = 25, 39.1%) and ‘joint decisions’ (patients: n = 86, 35.5%; HCPs: n = 25, 39.1%). Other elements of SDM and statements of the SDM questionnaires [24,27], such as ‘making clear that a decision needs to be made’ and ‘assessing patients’ preferred role in the decision-making process’, were not spontaneously written up (S2 Fig 1 in S2 Appendix).

Associations between patients’ awareness of the SDM concept and their characteristics (i.e., age, health literacy, gender, country, level of education, health status, treatment location, treatment duration, treatment lines, medical background and native language spoken in the hospital) were tested. For the following characteristics a strong association was found: a) *gender* (p = 0.007): more women had heard of the SDM concept, b) *country* (p < 0.001), c) *level of education* (p = 0.009), d) *health status* (p = 0.004): participants with a better health status indicated to have heard more of the SDM concept, and e) *medical background* (p = 0.001). Percentages between categories of these variables are presented in S2 Tables 1–6 in S2 Appendix. For HCPs, no strong association between their knowledge of SDM and their characteristics (i.e., age, gender, HCP type, workplace, years of experience, minutes per consultation, currently involved in research and currently involved in clinical guideline development) were found (S2 Table 7 in S2 Appendix).

### Current patient involvement

#### Involvement during consultations.

The SDM-Q-9 and SDM-Q-Doc questionnaires [24,27] were used to assess patients’ and HCPs’ experiences of patients’ current involvement in the decision-making process, respectively. All HCPs agreed that the advantages and disadvantages of treatment options are explained and that as HCP, they help their patient to understand all the information (i.e., step 4 and step 5 of the SDM questionnaires). On the other hand, around 30% of the participating patients disagreed that these steps were applied during their consultation (Fig 1). When comparing the questionnaires, SDM-Q scores were significantly higher in HCPs than in patients (t-test, p < 0.001), with patients having a lower median score (58, IQR: 42–71) than HCPs (78, IQR: 71–84), meaning that HCPs believe that during consultations they are applying the steps of the SDM process to a higher extent, than how it is perceived by patients (S3 Table 1 in S3 Appendix).

Patients were asked to what extent they felt involved during a) initial consultations following diagnosis and b) recent consultations. Most of the patients reported that they felt ‘somewhat’ to ‘extremely’ involved, both during initial (n = 368, 65.9%) and recent (n = 413,74.0%) consultations. More patients scored higher involvement at recent consultations (Table 3).

**Table 3:** Extent patients feel involved during consultations.

Involvement during consultations (n = 558)	Initial consultations following diagnosis, n (%)	Recent consultations, n (%)
Extremely	77 (13.8)	108 (19.4)
Quite a bit	185 (33.2)	205 (36.7)
Somewhat	106 (19.0)	100 (17.9)
A little bit	86 (15.4)	82 (14.7)
Not at all	96 (17.2)	51 (9.1)
I do not know	8 (1.4)	12 (2.2)

Most patients described the relationship with their treating physician as ‘very good’ (n = 317, 56.8%) or ‘good’ (n = 173, 31.0%). Some patients rated their relationship ‘neither bad nor good’ (n = 61, 10.9%). Few patients believed their relationship was ‘bad’ (n = 4, 0.7%) or ‘very bad’ (n = 3, 0.5%). This relationship was tested against involvement in recent consultations, SDM-Q-9 score and whether the HCP asked for the preferred way of receiving information or not, as described below.

Fisher’s exact test gave a significant p-value (p < 0.001), showing a difference in involvement in recent consultations depending on the relationship with the physician. When removing the ‘I do not know’ option, both variables can be considered ordinal, allowing the Spearman correlation to be calculated. The correlation was + 0.36 (associated p-value <0.001), indicating that patients who perceived more involvement also tended to report a better relationship with their physician (S3 Table 2 in S3 Appendix).

A one-way ANOVA showed a significant difference in SDM-Q-9 score across categories of the patient-physician relationship (p < 0.001). Patients reporting a better relationship with their physician tended to have higher SDM-Q-9 scores (S3 Table 3 in S3 Appendix).

A significant association was found between the relationship between the patient and the treating physician and whether the HCP asked patients about their preferred way of receiving information (Fisher’s exact test, p < 0.001; see (S3 Table 4 in S3 Appendix)).

HCPs were asked questions about the way they involve patients (Table 4). Most HCPs believe to ‘very often’ (n = 34, 38.2%) or ‘sometimes’ (n = 35, 39.3%) assess the preferred level of involvement of their patients.

**Table 4:** Questions on patients’ involvement and the assessment of preferred level of involvement answered by the participating HCPs.

Extent patient involvement (n = 89)	Initial consultations following diagnosis, n (%)	Later stages of the disease, n (%)	Transplant decisions, n (%)	Assessment patients’ preferred level of involvement (n = 89)	n (%)
Extremely	9 (10.1)	26 (29.2)	27 (30.3)	*Always*	12 (13.5)
Quite a bit	44 (49.4)	52 (58.4)	44 (49.4)	*Very often*	34 (38.2)
Somewhat	24 (27.0)	10 (11.2)	14 (15.7)	*Sometimes*	35 (39.3)
A little bit	11 (12.4)	1 (1.1)	3 (3.4)	*Rarely*	5 (5.6)
Not at all	1 (1.1)	0 (0.0)	1 (1.1)	*Never*	3 (3.4)

A positive correlation was observed between the extent to which HCPs involve patients at diagnosis and at later stage of the disease (r = 0.42; p-value<0.001). This indicates that HCPs who report high patient involvement at diagnosis also tend to report high patient involvement at later stages of the disease (S3 Fig 1 in S3 Appendix).

### Desired patient involvement

#### Reasons and barriers for involvement.

Most of the patients (n = 536, 96.1%) indicated they want to be involved in the decision-making processes. The main reasons were related to I) concern for their own body and health (n = 423, 78.9%), II) being the ones to experience the benefits, the side-effects and uncertainties of the treatments (n = 351, 65.5%) and III) involvement leading to a better decision for them (n = 319, 59.5%).

The top three barriers for involvement indicated by these patients, were I) lack of knowledge to be involved (n = 372, 69.4%), II) lack of understanding everything the HCP says (n = 331, 61.8%) and III) lack of willingness to talk about their disease, treatment, complaints and concerns (n = 234, 43.7%).

A minority of patients (n = 22, 3.9%) indicated not wanting to be involved. The main reasons were I) lack of knowledge to decide (n = 17, 77.3%), II) being used to the HCP deciding (n = 8, 36.4%) and III) not feeling it is their place to be involved (n = 55, 22.7%).

As reasons in favor of involvement, these patients indicated I) concern for their own body and health (n = 7, 31.8%), II) being the ones to experience the benefits, side-effects and uncertainties of the treatments (n = 6, 27.3%), and III) allowing a treatment decision to be in line with their personal values and preferences (n = 6, 27.3%).

Associations between patients’ characteristics and their desire to be involved were tested. Only the association between the country of residence and willingness to be involved was statistically significant (p = 0.026), meaning that the proportion of patients wanting to be involved or not varied across countries. Given the many variables and countries with zero counts, additional caution should be taken when interpreting this p-value, given the over and under representation of countries (S4 Table 1 and 2 in S4 Appendix).

All HCPs (n = 89) indicated that they believe patients should be involved in the decision-making process if they wanted to. The most indicated reasons were I) involvement of patients leading to improved compliance (n = 71, 79.8%), II) patients being the ones to experience the benefits, side-effects and uncertainties of the treatments (n = 64, 71.9%), and III) involvement of patients leading to better decisions (n = 61, 68.5%).

The most indicated possible barriers for patients to be involved were I) not wanting the responsibility to be involved (n = 71, 79.8%), II) lack of understanding everything the HCP says (n = 67, 75.3%), and III) lack of knowledge to be involved (n = 59, 66.3%).

Since all HCPs indicated that they believe patients should be involved, no associations could be tested.

#### Desired level of MM patients’ involvement during consultations.

The majority of patients indicated they would like to be ‘quite a bit’ or ‘extremely’ involved in both initial (n = 430, 77.1%) and recent (n = 447,80.1%) consultations (Table 5).

**Table 5:** Extent patients would have liked to be involved.

Desired involvement during consultations (n = 558)	Initial consultations following diagnosis, n (%)	Recent consultations, n (%)
Extremely	175 (31.4)	193 (34.6)
Quite a bit	255 (45.7)	254 (45.5)
Somewhat	65 (11.6)	70 (12.5)
A little bit	31 (5.6)	23 (4.1)
Not at all	14 (2.5)	7 (1.3)
I do not know	18 (3.2)	11 (2.0)

The correlations between patients’ current involvement (Table 3) and their desired involvement (Table 5) were found to be positive and significant both at diagnosis (r = 0.44; p < 0.001) and at recent consultations (r = 0.51; p < 0.001). For example, patients that indicated high current involvement at diagnosis tend to indicate high desired involvement at diagnosis.

Similarly, correlations between patients’ current involvement at diagnosis and recently (r = 0.66; p < 0.001) and desired involvement at diagnosis and recently (r = 0.76; p < 0.001) were also significant. For example, patients that indicated high current involvement at diagnosis tend to indicate high current involvement at recent consultations. Answers given by the participants are represented in S4 Fig 1 and S4 Table 3 in S4 Appendix.

#### Patients’ and treating physicians’ role in clinical decision-making.

**Fig 2:**
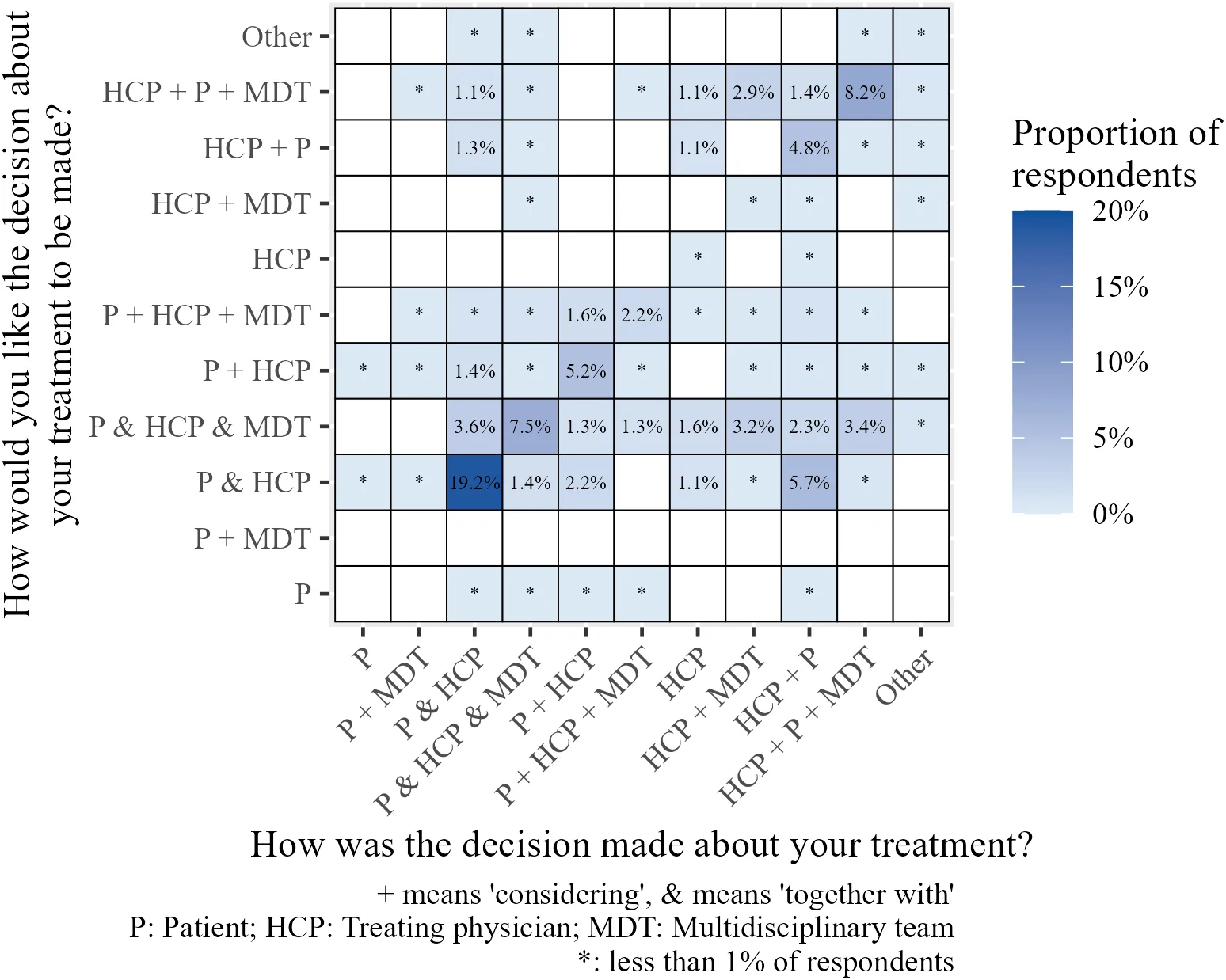
Comparing how the decision was made and how the patients would have liked the decision to be made. + means ‘considering’; & means ‘together with’; P: Patient; HCP: Treating physician; MDT: multidisciplinary team; *: less than 1% respondents, blank squares, 0% respondents. The diagonal elements sum up to 48%.

Patients were asked about their current and desired way of making decisions about their treatment. Different answer options were given, distinguishing who was involved (i.e., patient, treating physician, multidisciplinary team), and to what extent (i.e., considering the opinion, deciding together). Most patients (n = 156, 28.0%) indicated that the decisions are currently made together with the treating physician. A majority also indicated their desire to make the decisions a) together with the treating physician (n = 172, 30.8%), or b) together with the treating physician and the multidisciplinary team (n = 139, 24.9%). Fig 2 compares patients’ current and desired way of making decisions. It shows that 48% of all the patients found that the current way decisions are made is also their ideal way.

HCPs were also asked how they want decisions to be made. Most HCPs (n = 41, 46.1%) indicated that they want to make decisions together with the multidisciplinary team, considering the opinion of the patient.

## Discussion

### Unfamiliarity of the SDM concept among the survey participants

Our survey shows that many participants (316 patients (56.6%) and 25 HCPs (28.1%)) are not aware of the SDM concept, and that even if they have heard of the concept, their description of the concept did seldom entail elements related to actively assessing patients’ role and needs in the SDM process, as described as statements in the SDM questionnaires [24,27].

These findings align with those of other studies, showing still limited familiarity among MM patients and HCPs involved in MM with the SDM concept [20,21,30]. For example, in the interview study of Garvelink et al. [20] insights in HCPs’ perspectives on SDM were investigated. HCPs had the feeling to apply SDM, however when asked what SDM entailed, their definitions were not fully capturing the whole concept, in particular lacking elements on involvement of patients and the integration of patient preferences in the decision [20].

### Different perceptions on the current implementation of SDM in decision-making

Step 2 of the SDM questionnaire (i.e., ‘wanting to know how patients want to be involved’), was rated by patients in a very distributed way. 40% of the patients believed that the step of the treating physician wanting to know how patients want to be involved during the consultations, was not applied (i.e., rating towards disagreement); while 60% of the patients believed that the treating physician wanted to know (i.e., rating towards agreement). 80% of the HCPs on the other hand, believed that as treating physician they wanted to know how their patients want to be involved (i.e., rating towards agreement).

This shows the differences in perceptions between patients and HCPs concerning the extent to which the SDM steps are applied during consultations. HCPs rated the SDM steps systematically higher than patients, meaning that according to the participating HCPs, the SDM steps were applied to a higher extent during consultations.

Literature shows high intention among HCPs to engage patients in SDM but challenges its implementation [20,31,32]. The results of our survey show that there is willingness towards SDM and HCPs believe that SDM is taking place, however not all patients feel that all steps of the SDM are currently applied.

There was a statistically significant association between the SDM-Q-9 score and the relationship with the treating physician, showing a tendency towards higher scores when there was a better relationship. An interview study investigated how MM patients describe the development of trust in clinicians and the link with SDM. The findings showed that trust evolves throughout the treatment trajectory and that the impact on SDM is complex [19]. Trust has also been highlighted in literature as a facilitator for SDM [33–35]. This resonates with our survey findings suggesting the positive impact that trust in the HCPs can have on the implementation of SDM.

### Willingness among MM patients to be involved but challenges remain

Almost all patients (n = 536, 96.1%) indicated that they want to be involved, and all HCPs responded that they believe patients should be involved in decisions. This resonates with the findings of our interview study, showing that all participants agreed on involvement (to a certain extent) in decisions [21].

Another review assessed surveys with patients across different health conditions and showed that patients want and expect to be involved in decisions [30]. However, the interview study with patients with chronic hematological cancers of McCaughan et al. [22], had another conclusion. The results showed that many respondents express little or no desire to be actively involved in decisions. Some factors that prevented patients from participating actively were for example I) difficulties in getting and understanding information and II) feeling anxious, overwhelmed and unsupported [22].

The few patients (n = 22, 3.9%) that indicated not wanting to be involved in our survey indicated as main reason not knowing if they have enough knowledge to decide. This highlights room for improvement in helping patients to understand their disease and options, by changing the approach or type of information and support provided to patients, and the responsibility HCPs have in providing this information to patients in an accessible way. Questionnaires for patients to prepare their consultations and dedicated information for patients can be provided to help patients communicate their needs and preferences.

### Difference in involvement throughout the treatment trajectory

The survey shows that patients are more involved in treatment decisions concerning the later stages of their disease. Our survey revealed that patients desire to be more involved in initial decisions following diagnosis.

These findings are in line with the observations in the study of Nejati et al. [36], showing that the participating MM patients reported more SDM in a follow-up compared to baseline. In the study of Garvelink et al. [20] it was mentioned that SDM can be difficult in first line treatment decisions. Some reasons are the acute situations, in which there are directive guidelines and sometimes in these situations patients can be emotionally overwhelmed. Also, in the interview study conducted by McCaughan et al. [22] and in our interview study [21], the same results came out. When decisions had to be made fast and were urgent, involvement is sometimes circumvented.

### Preference in making decisions as patient and treating physician together

In the survey among patient and physicians in relapsed/refractory MM of Bylund et al. [23] questions on making the final decision where asked. 33% of the participating physicians believed that patients prefer that physicians make the final decision after considering patient’s opinion and 30% indicated that physicians and patients share the responsibility. Moreover, 40% of the patients preferred a shared responsibility and 25% indicated to prefer that physicians make the final decision, considering their opinion [23].

These findings are in line with our study as most patients indicated that they wanted the decisions to be made together with the treating physician (n = 172, 30.8%) or the treating physician and the multidisciplinary team (n = 139, 24.9%). Moreover, 156 patients (28.0%) indicated that decisions were made together with the treating physician. From the HCPs perspective, the option most indicated was ‘making the decision together with the multidisciplinary team, considering the opinion of the patient’.

On the contrary, two studies including hematological cancer patients, concluded that the preferred role in decision making of these patients was a passive or collaborative role, rather than an active role [37,38]. Reasons were related to the complex treatment plans and the perception of physicians being the experts [39]. Our survey revealed that the barrier indicated by most patients was related to the feeling of not having enough knowledge to decide. The variety shows the importance of assessing what is needed for each individual patient (e.g., assessing patients’ desired role in decisions, assessing patient preferences in terms of treatments and information needs) and act upon that.

### Participants’ characteristics did not reveal differences in involvement

The results regarding associations between willingness to be involved and participants’ characteristics showed that there was a significant difference for ‘country’, but caution is needed, given the over and under representation of countries. In the study of Tariman et al.[40], decisional preferences in older adults newly diagnosed with MM were determined. No statistically significant differences in decisional preferences across sociodemographic variables (e.g., gender, age, education, marital status, income and work status) were observed, in line with the findings of our survey.

These findings highlight the individuality of patients’ preferred way of being involved and strengthen the essential step of assessing what patients want and how patients want to be involved.

### Strengths and limitations of the survey

This study has several strengths. First, the survey was built upon a literature review and interviews [21]. Moreover, different stakeholders involved as part of a steering committee, and the patient organization (MPE), provided input and feedback throughout the whole process, enhancing the survey’s relevance and validity. The insights from the previously conducted studies, as well as input from different stakeholders involved, were used to design the surveys. The study was also pilot tested with patients and HCPs to ensure clarity and understandability of the questions. Second, the large and global sample size made it possible to conduct some sub-analyses, further strengthening the robustness of the findings. Third, the surveys consisted of some validated questionnaires, providing the opportunity to make comparisons with other studies where these questionnaires were used.

Regarding limitations, participants were recruited via the patient organization and participation in the study was voluntary, which may have led to selection bias towards participants with a better health and knowledge of the SDM concept. Moreover, the surveys were online, which could also lead to a selection bias towards participants with access to and confident with technology. Also, the survey was lengthy, which led to some people dropping out, and which may have led to survey fatigue and participants being less careful about what they respond.

Limitations concerning the design of the survey were observed. In most of the questions, predefined answer options were given, which may have influenced participants’ responses. Also, the surveys were answered globally but the distribution among the countries was not equal, making it hard to draw conclusions on differences between the countries. An exploratory comparison was performed between patients and the HCPs responses; however, because the data came from different groups, the results are not fully comparable. It is possible that HCPs do actively involve their patients but that it is not reflective of the HCPs of the patients that took part in the survey.

## Conclusion

The survey shows that most patients want to be involved and underlines the importance of starting from the patients and the patients’ needs and preferences concerning their involvement in MM treatment decision-making.

SDM shows to be relevant to help and guide patients and HCPs towards involvement. Still, the knowledge of SDM and the practical steps of SDM is low, among both patients and HCPs, in particular those elements related to the assessment of patients’ needs and preferences in SDM.

Awareness on how to implement the different SDM steps should be improved among both patients and HCPs. A mindset where patient involvement is done in a way that each individual patient feels comfortable is the way forward. HCPs should be aware that SDM is not just a checklist but a flexible and adaptable guideline toward patient involvement.

Moreover, building a trusting relationship between patients and HCPs can engage both parties to speak up and feel safe to share needs and preferences. Feeling that there is room to share uncertainties and fears can also stimulate those patients now feeling inadequate to be involved.

There is no one way that fits all patients in terms of involvement (i.e., the role in decision making, what is needed to feel involved, perceived and preferred levels of involvement). In the view of a patient-centred care, the individual patients’ needs and preferences should be prioritized. Next steps will be creating a frame with practical, stakeholder-informed recommendations that can help with the implementation of SDM in MM care, and guide and support HCPs and patients to listen and act with care.

## Supporting information

S1 Appendix Surveys. (DOCX)

S2 Appendix Knowledge of the ‘shared decision-making' (SDM) concept. (DOCX)

S3 Appendix Current patient involvement. (DOCX)

S4 Appendix Desired patient involvement. (DOCX)
