# Supplementary material for: Enhancing patient involvement through shared decision-making: Are we there yet in multiple myeloma? Findings from a cross-country survey
Source: PLoS One. 2026 Aug 28;21(8):e0356989. doi: 10.1371/journal.pone.0356989 (PMC13524276; doi:10.1371/journal.pone.0356989)
Supplement: Supplementary file 1 [file pone.0356989.s001.docx]

S1. Appendix 1 – Surveys

Patient survey

**Survey on European myeloma patients’ and healthcare professionals' perspectives and preferences on the role of patients in treatment and care decision-making**

This study aims to:

1) understand your perspectives on **how treatment and care decisions are currently made in myeloma**.
2) identify what you think is, and should be, **the role of patients and healthcare professionals** in the decision-making processes.
3) investigate what **patient involvement in decision-making on treatment and care** means and how this can be achieved and optimised.
4) formulate **recommendations** **on** how to improve the decision-making processes and how to create optimal patient involvement.

Results are intended to inform improved processes for patient involvement in treatment and care decision-making.

**INFORMATION SHEET**

More information of the study which aims to find out perceptions regarding decision making can be found in the information sheet.

**PARTS OF THE SURVEY AND GENERAL INFORMATION**

Thank you for your participation!

The survey will consist of five parts:

- Part 1: Personal characteristics
- Part 2: Disease and treatment related information
- Part 3: Patient involvement
- Part 4: Sources of information
- Part 5: Your role in decision-making and relationship with healthcare professionals

Please complete all five parts as it is important for the research analysis and conclusions to have fully completed surveys.

The answers to the following questions will be summarised as group characteristics. Only anonymous data will be processed.

If a question is unclear or you would like additional information, please email charlotte.verbeke@kuleuven.be.

This survey will take approximately 20 minutes to complete.

**SURVEY**

1. **PERSONAL CHARCTERISTICS**
   - Have you received a confirmed diagnosis of myeloma (also known as multiple myeloma)?
     - Yes
     - No
     - *If answer no: ”Since you did not receive a confirmed diagnosis of myeloma, you do not fall in the scope, and it is not possible to participate in this study. However, we thank you for your interest.”*
   - What is your gender?
     - Male
     - Female
     - Non-binary/gender non-conforming
     - Other, please specify:
     - Prefer not to say
   - What is your age?
     - *“As you are under 18 years old, you do not fall in the scope, and it is not possible to participate in this study. However, we thank you for your interest.”*
   - Which country do you live in?
   - Is your native language spoken at the hospital?
     - Yes
     - No
   - What is your highest level of education?
     - I did not finish high school
     - High school
     - Bachelor's degree
     - Master’s degree
     - Doctorate
     - Other, please specify:
   - Do you have a medical background (e.g., doctor, nurse, pharmacist, health care personnel)?
     - Yes
     - No
     - I am not sure
   - What is your current employment status? (Multiple options possible)
     - Student
     - Full-time
     - Part-time
     - Employed but currently not working due to my disease
     - Unemployed
     - Self-employed
     - Retired
     - Prefer not to say
     - Other, please specify
   - With whom do you usually go to your consultations? (Multiple options possible)
     - Alone
     - With my partner
     - With my child
     - With a family member
     - With a friend
     - Other
   - How often do you have someone (like a family member, friend, hospital/clinic worker, or caregiver) help you read hospital materials?
     - Always
     - Often
     - Sometimes
     - Occasionally
     - Never
   - How confident are you filling out medical forms by yourself?
     - Extremely
     - Quite a bit
     - Somewhat
     - A little bit
     - Not at all
   - How often do you struggle when learning about your medical condition because you have difficulty understanding written information?
     - Always
     - Often
     - Sometimes
     - Occasionally
     - Never
2. **DISEASE AND TREATMENT RELATED INFORMATION**
   - How would you currently rate your health on a scale from 0-100 (with 0 = poor health and 100 = excellent health)?
   - I am currently being treated for my myeloma in a:
     - University hospital/academic centre
     - General hospital
     - I am not sure
     - Other, namely:
   - At what age were you diagnosed with myeloma?
   - Which treatment(s) are you currently undergoing for your myeloma? (Multiple options possible)
     - Chemotherapy
     - Radiotherapy
     - Immunotherapy
     - Treatment with corticosteroids
     - Targeted therapy
     - Stem cell transplantation
     - Maintenance treatment (e.g., a low dose to maintain your response to treatment, usually with lenalidomide)
     - General care (e.g., regular visits, blood tests)
     - No treatment
     - Other, please specify:
   - How much time has passed since the last treatment decision was made (e.g., changing the type of treatment, changing the mode of administration, changing the frequency of receiving treatment, adaption of dosage)?
     - Months ______
     - Years	______
   - How many treatment lines* have you received up until now, including your current treatment:
     - 1 line*
     - 2 lines*
     - 3 lines*
     - 4 lines*
     - 5 lines*
     - 5^th^ line*
     - More, namely:
     - I am not sure

**Note:*

- *A treatment ‘line’ refers to the treatment or combination of treatments prescribed to you until your disease is under control (you reach remission or stable phase). That line of treatment may include a combination of 2 or 3 anti-myeloma medicines given at the same time. A stem cell transplant is considered a separate treatment line.*
  - Which of the following treatments have you received up until now, including your current treatment ? (multiple answers possible).
    - Chemotherapy
    - Radiotherapy
    - Immunotherapy
    - Treatment with corticosteroids
    - Targeted therapy
    - Stem cell transplant
    - Maintenance treatment
      - **When stem cell transplant is not indicated:** Was the option of undergoing a stem cell transplant presented to you?
        - Yes
        - No
        - I am waiting to undergo a stem cell transplant
        - I am not sure
      - **When stem cell transplantation indicated:** How many stem cell transplants did you undergo?
        - I am waiting to undergo a stem cell transplant
        - 1
        - 2
        - More, please specify:
- **IF ANSWER 1 or I am waiting to undergo a stem cell transplant**: Did you get the option of not undergoing the stem cell transplant?
  - Yes
  - No
  - I am not sure
- **IF ANSWER 1:** Was the option of undergoing a second transplant presented to you?
  - Yes
  - No
  - I am not sure
- **IF ANSWER 2 OR MORE**: Did you get the option of not undergoing the first stem cell transplant?
  - Yes
  - No
  - I am not sure
- **IF ANSWER 2 OR MORE:** Did you get the option of not undergoing the second stem cell transplant?
  - Yes
  - No
  - I am not sure
- **IF ANSWER MORE:** Did you get the option of not undergoing the third (and following) stem cell transplant(s)?
  - Yes
  - No
  - I am not sure

3. **PATIENT INVOLVEMENT**

*YOUR OPINION ON THE LEVEL AND WAY TO BE INVOLVED IN THE DECISION-MAKING PROCESSES*

- Do you want to be involved in the decision-making process regarding your myeloma treatment?
  - Yes
  - No
- *IF ANSWER YES:* Why do you want to be involved in decision-making concerning your treatment and care? (Multiple options possible)
  - It is my body and health
  - My opinion is as important as that of my healthcare professional(s)*
  - As the patient, I will experience the benefits, the side-effects and uncertainties of the treatment
  - My involvement may lead to a better decision for me as a patient
  - My involvement may lead to better satisfaction
  - My involvement may lead to an improved compliance
  - It empowers me
  - It makes me feel more confident
  - It gives me control
  - It allows a treatment decision to be in line with my personal values and preferences
  - I will receive better care if I have a say in the decisions to be made
  - You make better decisions together than alone
  - I have enough knowledge to be able to talk to the healthcare professionals
  - Other, please specify:
  - How would you rate the options you indicated? Please click on your indicated options and drag them in the desired order.

*Note: ranking of the different options as 1: being the most important to 14: being the least important*

**Note: Healthcare professionals (HCPs): all members of the multidisciplinary team (e.g., haematologists, oncologists, nurses).*

- IF ANSWER YES: What could be barriers for patients to be involved in the decision-making concerning their treatment and care? (Multiple options possible)
  - Some patients do not have enough knowledge to be involved
  - Some patients do not want the responsibility to be involved
  - Some patients feel that their treating physician is not open to patient involvement
  - Some patients are too ill to be involved
  - Some patients do not like to talk about their disease, treatment, complaints and concerns
  - Some patients feel time pressured during the appointment
  - Some patients have (too) little time to think about their personal values and preferences
  - Some patients do not understand everything the healthcare professional says
  - There is too little patient focused information available for patient to support decision-making
  - The clinical setting (e.g., busy or noisy ward, disrupted consultations) does not facilitate a comfortable discussion
  - Other, please specify:
- How would you rate the options you indicated? Please click on your indicated options and drag them in the desired order.

*Note: ranking of the different options as 1: being the most important to 11: being the least important*

- *IF ANSWER NO*: Why do you not want to be involved? (Multiple options possible)
  - I do not know if I have enough knowledge to decide
  - I do not feel it is my place as the patient to be involved
  - I do not want the responsibility to decide
  - I feel too ill to be involved
  - I do not like to talk about my disease, treatment, complaints or concerns
  - I feel time pressured during the appointment
  - I have (too) little time to think about my personal values and preferences
  - I do not understand everything the healthcare professional says
  - I sometimes have the feeling that my healthcare professional does not take me seriously
  - My healthcare professional gives me too little patient focused information
  - I am sometimes afraid that an inferior decision will be made if I participate
  - I am used to the healthcare professional deciding
  - I do not think it is that important to decide together with the healthcare professional
  - I have the feeling that actively participating is not getting me anywhere
  - My healthcare professional does not ask me if I want to be involved
  - The clinical setting (*e.g., busy or noisy ward, disrupted consultations*) does not allow me to feel comfortable enough to share my preferences
  - Other, please specify:
- How would you rate the options you indicated? Please click on your indicated options and drag them in the desired order.

*Note: ranking of the different options as 1: being the most important to 17: being the least important*

**Note: Healthcare professionals (HCPs): all members of the multidisciplinary team (e.g., haematologists, oncologists, nurses).*

- IF ANSWER NO: What could be possible reasons why patient would like to be involved in the decision-making concerning their treatment and care? (Multiple options possible)
  - It is their body and health
  - The opinion of the patients is as important as the opinion of the healthcare professionals
  - Patients will experience the benefits, side-effects and uncertainties of the treatment
  - Involving patients may lead to a better decision for the patient
  - Involving patients may lead to better satisfaction
  - Involving patient may lead to an improved compliance
  - It empowers the patient
  - It makes patients feel more confident
  - It gives patients control
  - It allows a treatment decision to be in line with the personal values and preferences of the patient
  - You make better decisions together than alone
  - Patients know quite a lot about the care they need
  - Other, please specify:
- How would you rate the options you indicated? Please click on your indicated options and drag them in the desired order.

*Note: ranking of the different options as 1: being the most important to 13: being the least important*

- Have you previously heard about the concept of shared decision-making?
- Yes
  - No
    - IF ANSWER NO: What do you think is/are essential steps for decision-making with your treating physician*? (Multiple options possible)
      - My treating physician makes clear that a decision needs to be made
      - My treating physician asks how I want to be involved in the decision-making process
      - My treating physician tells me that there are different options for treating my medical condition
      - My treating physician precisely explains the advantages and disadvantages of the treatment options
      - My treating physician helps me to understand all the information
      - My treating physician asks me which treatment option I prefer
      - My treating physician and I thoroughly weigh the different treatment options
      - My treating physician and I select a treatment option together
      - My treating physician and I reach an agreement on how to proceed
      - Other, namely:
      - None of the above

**Note: Treating physician: the physician making decisions concerning the treatment. Depending on the decision, this can for example be your haematologist or your oncologist*

- IF ANSWER YES:
- How would you describe shared decision-making in your own words?
- What do you think is essential for shared decision-making? (Multiple options possible)
  - My treating physician* makes clear that a decision needs to be made
  - My treating physician asks how I want to be involved in the decision-making process
  - My treating physician tells me that there are different options for treating my medical condition
  - My treating physician precisely explains the advantages and disadvantages of the treatment options
  - My treating physician helps me to understand all the information
  - My treating physician asks me which treatment option I prefer
  - My treating physician and I thoroughly weigh the different treatment options
  - My treating physician and I select a treatment option together
  - My treating physician and I reach an agreement on how to proceed
  - Other, namely:
  - None of the above

** Note: Treating physician: the physician making decisions concerning the treatment. Depending on the decision, this can for example be your haematologist or your oncologist*

*YOUR OPINION ON THE CURRENT TREATMENT DECISION-MAKING PROCESS*

*Please read the statements below and indicate to what extent you agree with them. Think back* ***at a recent consultation*** *with your treating physician* where a decision (e.g., decision to stop or change therapy, decision to go for a transplant, decision to go into a clinical trial) had to be made concerning your myeloma treatment (the decision to discontinue or not start a treatment can also be seen as a treatment option).*

** Note: Treating physician: the physician making decisions concerning the treatment. Depending on the decision, this can for example be your haematologist or your oncologist*

- My treating physician made clear that a decision needs to be made
  - Completely disagree
  - Strongly disagree
  - Somewhat disagree
  - Somewhat agree
  - Strongly agree
  - Completely agree
- My treating physician wanted to know exactly how I want to be involved in making the decision
  - Completely disagree
  - Strongly disagree
  - Somewhat disagree
  - Somewhat agree
  - Strongly agree
  - Completely agree
- My treating physician told me that there are different options for treating my medical condition
  - Completely disagree
  - Strongly disagree
  - Somewhat disagree
  - Somewhat agree
  - Strongly agree
  - Completely agree
- My treating physician precisely explained the advantages and disadvantages of the treatment options
  - Completely disagree
  - Strongly disagree
  - Somewhat disagree
  - Somewhat agree
  - Strongly agree
  - Completely agree
- My treating physician helped me understand all the information
  - Completely disagree
  - Strongly disagree
  - Somewhat disagree
  - Somewhat agree
  - Strongly agree
  - Completely agree
- My treating physician asked me which treatment option I prefer
  - Completely disagree
  - Strongly disagree
  - Somewhat disagree
  - Somewhat agree
  - Strongly agree
  - Completely agree
- My treating physician and I thoroughly weighed the different treatment options
  - Completely disagree
  - Strongly disagree
  - Somewhat disagree
  - Somewhat agree
  - Strongly agree
  - Completely agree
- My treating physician and I selected a treatment option together
  - Completely disagree
  - Strongly disagree
  - Somewhat disagree
  - Somewhat agree
  - Strongly agree
  - Completely agree
- My treating physician and I reached an agreement on how to proceed
  - Completely disagree
  - Strongly disagree
  - Somewhat disagree
  - Somewhat agree
  - Strongly agree
  - Completely agree

*YOUR OPINION ON YOUR IDEAL LEVEL OF INVOLVEMENT IN TREATMENT DECISION-MAKING*

*To complete the following questions, please consider* ***your initial consultations with your treating physician following diagnosis.***

- To what extent **were you involved** in the decision-making processes?
  - Extremely
  - Quite a bit
  - Somewhat
  - A little bit
  - Not at all
  - I do not know
- To what extent **would you have liked to be involved** in the decision-making processes?
  - Extremely
  - Quite a bit
  - Somewhat
  - A little bit
  - Not at all
  - I do not know

*To complete the following questions, please consider* ***a recent consultation*** *with your treating physician where a decision (e.g., decision to stop or change therapy, decision to go for a transplant, decision to go into a clinical trial) had to be made concerning your myeloma treatment.*

- To what extent **were you involved** in the decision-making processes?
  - Extremely
  - Quite a bit
  - Somewhat
  - A little bit
  - Not at all
  - I do not know
- To what extent **would you have liked to be** involved in the decision-making processes?
  - Extremely
  - Quite a bit
  - Somewhat
  - A little bit
  - Not at all
  - I do not know

4. **SOURCES OF INFORMATION**

*YOUR OPINION ON THE INFORMATION SHARING IN TREATMENT DECISION-MAKING*

- From which **source** **did you receive information** concerning your disease and treatment options? (Multiple options possible)
  - My healthcare professional(s) *

🡪 if indicated: please specify from which healthcare professional(s)

- Haematologist
- Oncologist
- Haemato-oncologist
- Nurse specialist
- Nurse
- Other, please specify:
- Patient organizations, patient support groups
- Pharmaceutical companies
- Healthcare authorities (such as the European Medicines Agency, national healthcare agencies)
- Search engine (e.g., Google, Bing, Yahoo!, ChatGPT)
- Other, please specify:
- None
- IF ANSWERED MY HEALTHCARE PROFESSIONALS: Via **which channel(s) did you receive information** concerning your disease and treatment options **from your healthcare processional(s)**? (Multiple options possible)
  - Via oral discussions
  - Information leaflets
  - Videos
  - Social media (e.g., YouTube, Instagram, Facebook)
  - Websites
  - Other, please specify:
- IF ANSWERED PATIENT ORGANIZATIONS, PATIENT SUPPORT GROUPS: Via **which channel(s) did you receive information** concerning your disease and treatment options **from patient organization and patient support groups**? (Multiple options possible)
  - Gatherings (meetings, conferences)
  - Information leaflets
  - Videos
  - Social media (e.g., YouTube, Instagram, Facebook)
  - Websites
  - Other, please specify:
- IF ANSWERED PHARMACEUTICAL COMPANIES: Via **which channel(s) did you receive information** concerning your disease and treatment options **from pharmaceutical companies?** (Multiple options possible)
  - Information leaflets
  - Videos
  - Social media (e.g., YouTube, Instagram, Facebook)
  - Websites
  - Other, please specify:
- IF ANSWERED HEALTHCARE AUTHORITIES (SUCH AS EUROPEAN MEDICINES AGENCY): Via **which channel(s) did you receive information** concerning your disease and treatment options **from Healthcare authorities (such as the European Medicines Agency, national healthcare agencies)** ? (Multiple options possible)
  - Information leaflets
  - Videos
  - Social media (e.g., YouTube, Instagram, Facebook)
  - Websites
  - Other, please specify:
- From which source **would you like to** receive information concerning your disease and treatment options? (Multiple options possible)
  - My healthcare professional(s)

🡪 if indicated: please specify from which healthcare professional(s)

- Haematologist
- Oncologist
- Haemato-oncologist
- Nurse specialist
- Nurse
- Other, please specify:
- Patient organizations, patient support groups
- Pharmaceutical companies
- Healthcare authorities (such as the European Medicines Agency, national healthcare agencies)
- Search engine (e.g., Google, Bing, Yahoo!, ChatGPT)
- Other, please specify:
- None
- IF ANSWERED MY HEALTHCARE PROFESSIONALS: Via **which channel(s) would you like to receive information** concerning your disease and treatment options **from your healthcare processional(s)**? (Multiple options possible)
  - Via oral discussions
  - Information leaflets
  - Videos
  - Social media (e.g., YouTube, Instagram, Facebook)
  - Websites
  - Other, please specify:
- IF ANSWERED PATIENT ORGANIZATIONS, PATIENT SUPPORT GROUPS: Via **which channel(s) would you like to receive information** concerning your disease and treatment options **from patient organizations and patient support groups**? (Multiple options possible)
  - Gatherings (meetings, conferences)
  - Information leaflets
  - Videos
  - Social media (e.g., YouTube, Instagram, Facebook)
  - Websites
  - Other, please specify:
- IF ANSWERED PHARMACEUTICAL COMPANIES: Via **which channel(s) would you like to receive information** concerning your disease and treatment options **from pharmaceutical companies**? (Multiple options possible)
  - Information leaflets
  - Videos
  - Social media (e.g., YouTube, Instagram, Facebook)
  - Websites
  - Other, please specify:
- IF ANSWERED HEALTHCARE AUTHORITIES (SUCH AS EUROPEAN MEDICINES AGENCY): Via **which channel(s) would you like to receive information** concerning your disease and treatment options **from Healthcare authorities (such as the European Medicines Agency, national healthcare agencies)?** (Multiple options possible)
  - Information leaflets
  - Videos
  - Social media (e.g., YouTube, Instagram, Facebook)
  - Websites
  - Other, please specify:
- Considering your recent consultations, did a healthcare professional* ask how you would like to receive information concerning your disease and treatment options?
  - Yes
  - No
  - I am not sure
  - If yes, which healthcare professional(s)*?
    - Haematologist
    - Oncologist
    - Haemato-oncologist
    - Nurse specialist
    - Nurse
    - Other, please specify:

**Note: Healthcare professionals (HCPs): all members of the multidisciplinary team (e.g., haematologists, oncologists, nurses).*

5. **YOUR ROLE IN DECISION-MAKING AND RELATIONSHIP WITH HEALTHCARE PROFESSIONALS**

*To complete the following questions, please consider a recent consultation with your treating physician where a decision (e.g., decision to stop or change therapy, decision to go for a transplant, decision to go into a clinical trial) had to be made concerning your myeloma treatment.*

- Who made the final decision about your treatment?
  - I made the decision **myself,** **without considering** the opinion of **my treating physician**
  - I made the decision **myself**, **without considering** the opinion of **my treating physician** and the **multidisciplinary team**
  - I made the decision **myself**, **considering** the opinion of **my treating physician**
  - I made the decision **myself,** **considering** the opinion of **my treating physician** and the one of the **multidisciplinary team**
  - My **treating physician and I** made the decision **together**
  - My **treating physician**, the **multidisciplinary team** **and I** made the decision **together**
  - My **treating physician** made the decision, **considering my opinion**
  - My **treating physician** made the decision together with the **multidisciplinary team**, **considering my opinion**
  - My **treating physician** made the decision **himself/herself**, **without considering my opinion**
  - My **treating physician** made the decision together with the **multidisciplinary team**, **without considering my opinion**
  - Other, please specify:
- How would you have liked a decision about your treatment to be made?
  - I want to make the decision **myself**, **without considering** the opinion of **my treating physician**
  - I want to make the decision **myself**, **without considering** the opinion of **my treating physician** and **the multidisciplinary team**
  - I want to make the decision **myself**, **considering** the opinion of **my treating physician**
  - I want to make the decision **myself**, **considering** the opinion of **my treating physician** and the one of **the multidisciplinary team**
  - I want to make the decision **together with my treating physician**
  - I want to make the decision **together with my treating physician** and the **multidisciplinary team**
  - I want **my treating physician** to make the decision, **considering my opinion**
  - I want **my treating physician** together with the **multidisciplinary team** to make the decision, **considering my opinion**
  - I want **my treating physician** to make the decision **himself/herself**, **without considering my opinion**
  - I want **my treating physician** together with the **multidisciplinary team** to make the decision, **without considering my opinion**
  - Other, please specify:
- How would you describe your relationship with your current treating physician?
  - Very bad
  - Bad
  - Neither bad nor good
  - Good
  - Very good
  - What do you think is needed for you to achieve your ideal role in (treatment) decision-making processes?
  - Which healthcare professional(s)* is/are currently involved in your (treatment) decision-making processes? (Multiple options possible)
    - Haematologist
    - Oncologist
    - Haemato-oncologist
    - Nurse specialist
    - Nurse
    - Other, please specify:
  - Which healthcare professional(s)* do you think can have a (more prominent) role than presently in the (treatment) decision-making processes? (Multiple options possible)
    - Haematologist
    - Oncologist
    - Haemato-oncologist
    - Nurse specialist
    - Nurse
    - Other, please specify:
    - None
  - In which steps of the decision-making process do you think **haematologists** can have a (more prominent) role than presently? (Multiple options possible)
    - *Making clear that a decision needs to be made*
    - *Wanting to know exactly how I want to be involved in the decision-making process*
    - *Telling that there are different options for treating my medical condition*
    - *Precisely explaining the advantages and disadvantages of the treatment options*
    - *Helping me understand all the information*
    - *Asking me which treatment option I prefer*
    - *Thoroughly weighing the different treatment options*
    - *Selecting a treatment option together*
    - *Reaching an agreement on how to proceed*
    - *Other, please specify:*
    - *All of the above*
    - *None of the above*
  - In which steps of the decision-making process do you think **oncologists** can have a (more prominent) role than presently? (Multiple options possible)
    - *Making clear that a decision needs to be made*
    - *Wanting to know exactly how I want to be involved in the decision-making process*
    - *Telling that there are different options for treating my medical condition*
    - *Precisely explaining the advantages and disadvantages of the treatment options*
    - *Helping me understand all the information*
    - *Asking me which treatment option I prefer*
    - *Thoroughly weighing the different treatment options*
    - *Selecting a treatment option together*
    - *Reaching an agreement on how to proceed*
    - *Other, please specify:*
    - *All of the above*
    - *None of the above*
  - In which steps of the decision-making process do you think **haemato-oncologists** can have a (more prominent) role than presently? (Multiple options possible)
    - *Making clear that a decision needs to be made*
    - *Wanting to know exactly how I want to be involved in the decision-making process*
    - *Telling that there are different options for treating my medical condition*
    - *Precisely explaining the advantages and disadvantages of the treatment options*
    - *Helping me understand all the information*
    - *Asking me which treatment option I prefer*
    - *Thoroughly weighing the different treatment options*
    - *Selecting a treatment option together*
    - *Reaching an agreement on how to proceed*
    - *Other, please specify:*
    - *All of the above*
    - *None of the above*
  - In which steps of the decision-making process do you think **nurse specialists** can have a (more prominent) role than presently? (Multiple options possible)
    - *Making clear that a decision needs to be made*
    - *Wanting to know exactly how I want to be involved in the decision-making process*
    - *Telling that there are different options for treating my medical condition*
    - *Precisely explaining the advantages and disadvantages of the treatment options*
    - *Helping me understand all the information*
    - *Asking me which treatment option I prefer*
    - *Thoroughly weighing the different treatment options*
    - *Selecting a treatment option together*
    - *Reaching an agreement on how to proceed*
    - *Other, please specify:*
    - *All of the above*
    - *None of the above*
  - In which steps of the decision-making process do you think **nurses** can have a (more prominent) role than presently? (Multiple options possible)
    - *Making clear that a decision needs to be made*
    - *Wanting to know exactly how I want to be involved in the decision-making process*
    - *Telling that there are different options for treating my medical condition*
    - *Precisely explaining the advantages and disadvantages of the treatment options*
    - *Helping me understand all the information*
    - *Asking me which treatment option I prefer*
    - *Thoroughly weighing the different treatment options*
    - *Selecting a treatment option together*
    - *Reaching an agreement on how to proceed*
    - *Other, please specify:*
    - *All of the above*
    - *None of the above*
  - In which steps of the decision-making process do you think **the other people you** **indicated in the previous question** can have a (more prominent) role than presently? (Multiple options possible)
    - *Making clear that a decision needs to be made*
    - *Wanting to know exactly how I want to be involved in the decision-making process*
    - *Telling that there are different options for treating my medical condition*
    - *Precisely explaining the advantages and disadvantages of the treatment options*
    - *Helping me understand all the information*
    - *Asking me which treatment option I prefer*
    - *Thoroughly weighing the different treatment options*
    - *Selecting a treatment option together*
    - *Reaching an agreement on how to proceed*
    - *Other, please specify:*
    - *All of the above*
    - *None of the above*

*FEEDBACK QUESTIONS:*

- What did you think about the length of the survey?
  - Way too long
  - Too long
  - Manageable
  - Just right
- How easy or difficult was it for you to understand the questions?
  - Very easy
  - Easy
  - Difficult
  - Very difficult
- How easy or difficult was it for you to answer the questions?
  - Very easy
  - Easy
  - Difficult
  - Very difficult
- Do you have any suggestions for improving the survey? If so, please describe them here. If you do not have any remarks, please enter a "/" or type "NA".

HCP survey

**Survey on myeloma patients’ and healthcare professionals’ perspectives and preferences on the role of patients in treatment and care decision-making**

This study aims to:

5) understand your perspectives on **how treatment and care decisions are currently made in myeloma**.
6) identify what you think is, and should be, **the role of patients and healthcare professionals** in the decision-making processes.
7) investigate what **patient involvement in decision-making on treatment and care** means and how this can be achieved and optimised.
8) formulate **recommendations** on how to improve decision-making processes and how to create optimal patient involvement.

Results are intended to inform improved processes for patient involvement in treatment and care decision-making.

**INFORMATION SHEET**

More information of the study which aims to find out perceptions regarding decision making can be found in the information sheet.

**PARTS OF THE SURVEY AND GENERAL INFORMATION**

Thank you for your participation!

The survey will consist of four parts:

- Part 1: Personal characteristics
- Part 2: Patient involvement
- Part 3: Sources of information
- Part 4: Ideal role for patients and healthcare professionals

Please complete all four parts as it is important for the research analysis and conclusions to have fully completed surveys.

The answers to the following questions will be summarised as group characteristics. Only anonymous data will be processed.

If a question is unclear or you would like additional information, please email charlotte.verbeke@kuleuven.be.

This survey will take approximately 10 minutes to complete.

**SURVEY**

6. **PERSONAL CHARACTERISTICS**
   - What is your gender?
     - Male
     - Female
     - Non-binary/gender non-conforming
     - Other, please specify
     - Prefer not to say
   - What is your age?
   - Which country do you live in?
   - Are you a healthcare professional currently involved in myeloma treatment?
     - Yes
     - No
       - *If answers no: Since you are currently not involved in myeloma treatment, it is not possible to participate in this study. However, we thank you for your interest.*
   - I am a/an:
     - Haematologist
     - Oncologist
     - Haemato-oncologist
     - Nurse specialist
     - Nurse
     - Other healthcare professional, please specify:
     - None of the above
       - *If answer none of the above: Since you are not a healthcare professional, it is not possible to participate in this study. However, we thank you for your interest.*
   - I am currently working in a:
     - University hospital/academic centre
     - General hospital
     - Other, namely:
   - How many years of experience do you have in treating or caring for myeloma patients (including training and specialisation)?
   - How many minutes do you spend on average per consultation with a myeloma patient when a decision has to be made?
   - Are you currently involved in research (e.g. clinical trials, implementation healthcare research, social research)?
     - Yes
     - No
   - Are you currently involved in the development of clinical practice guidelines for myeloma?
     - Yes
     - No
7. **PATIENT INVOLVEMENT**

*YOUR OPINION ON THE LEVEL AND WAY TO INVOLVE PATIENTS IN THE DECISION-MAKING PROCESSES*

- Do you think patients should be involved in the decision-making process, if they want to?
  - Yes
  - No
- IF ANSWER YES: Why do you think patients should be involved? (Multiple options possible)
  - It is their body and health
  - The opinion of the patients is as important as the opinion of the healthcare professionals
  - Patients will experience the benefits, side-effects and uncertainties of the treatment
  - Involving patients may lead to a better decision for the patient
  - Involving patients may lead to better satisfaction
  - Involving patient may lead to an improved compliance
  - It empowers the patient
  - It makes patients feel more confident
  - It gives patients control
  - It allows a treatment decision to be in line with the personal values and preferences of the patient
  - You make better decisions together than alone
  - Patients know quite a lot about the care they need
  - Other, please specify:
  - How would you rate the options you indicated? Please click on your indicated options and drag them in the desired order.

*Note: ranking of the different options as 1: being the most important to 13: being the least important*

- IF ANSWER YES: What could be barriers for patients to be involved in the decision-making concerning their treatment and care? (Multiple options possible)
  - Some patients do not have enough knowledge to be involved
  - Some patients do not want the responsibility to be involved
  - Some patients feel that their treating physician is not open to patient involvement
  - Some patients are too ill to be involved
  - Some patients do not like to talk about their disease, treatment, complaints and concerns
  - Some patients feel time pressured during the appointment
  - Some patients have (too) little time to think about their personal values and preferences
  - Some patients do not understand everything the healthcare professional says
  - There is too little patient focused information available for patient to support decision-making
  - The clinical setting (e.g., busy or noisy ward, disrupted consultations) does not facilitate a comfortable discussion
  - Other, please specify:
- How would you rate the options you indicated? Please click on your indicated options and drag them in the desired order.

*Note: ranking of the different options as 1: being the most important to 11: being the least important*

- IF ANSWER NO: Why do you think patients should not be involved? (Multiple options possible)
  - I do not know if patients have enough knowledge to be involved
  - I do not know if it is the responsibility of patients to be involved
  - Some patients do not want the responsibility to be involved
  - Some patients are too ill to be involved
  - Some patients do not like to talk about their disease, treatment, complaints and concerns
  - Some patients feel time pressured during the appointment
  - Some patients have (too) little time to think about their personal values and preferences
  - Some patients do not understand everything the healthcare professional says
  - There is too little patient focused information available for patient to support decision-making
  - Some patients are used to the fact that their treating physician decides
  - I have the feeling that actively involving patients is not getting us anywhere
  - The clinical setting (*e.g., busy or noisy ward, disrupted consultations*) does not facilitate a comfortable discussion
  - Involving patients makes me look indecisive or insecure
  - Other, please specify:
- How would you rate the options you indicated? Please click on your indicated options and drag them in the desired order.

*Note: ranking of the different options as 1: being the most important to 14: being the least important*

- IF ANSWER NO: What could be possible reasons why patient would like to be involved in the decision-making concerning their treatment and care? (Multiple options possible)
  - It is their body and health
  - The opinion of the patients is as important as the opinion of the healthcare professionals
  - Patients will experience the benefits, side-effects and uncertainties of the treatment
  - Involving patients may lead to a better decision for the patient
  - Involving patients may lead to better satisfaction
  - Involving patient may lead to an improved compliance with the treatment decision
  - It empowers the patient
  - It makes patients feel more confident
  - It gives patients control
  - It allows a treatment decision to be in line with the personal values and preferences of the patient
  - You make better decisions together than alone
  - Patients know quite a lot about the care they need
  - Other, please specify:
- How would you rate the options you indicated? Please click on your indicated options and drag them in the desired order.

*Note: ranking of the different options as 1: being the most important to 13: being the least important*

- Have you previously heard about the concept of shared decision-making?
  - Yes
  - No
    - IF ANSWER NO: What do you think is/are essential steps for decision-making between you and the patient? (Multiple options possible)
      - You make clear that a decision needs to be made
      - You ask how the patient wants to be involved in the decision-making process
      - You tell the patient that there are different options for treating his/her medical condition
      - You precisely explain the advantages and disadvantages of the treatment options to the patient
      - You help the patient to understand all the information
      - You ask the patient which treatment option he/she prefers
      - You and the patient thoroughly weigh the different treatment options
      - You and the patient select a treatment option together
      - You and the patient reach an agreement on how to proceed
      - Other, namely:
      - None of the above
    - IF ANSWER YES:
- How would you describe shared decision-making in your own words?
- What do you think is essential for shared decision-making? (Multiple options possible)
  - You make clear that a decision needs to be made
  - You ask how the patient wants to be involved in the decision-making process
  - You tell the patient that there are different options for treating his/her medical condition
  - You precisely explain the advantages and disadvantages of the treatment options to the patient
  - You help the patient to understand all the information
  - You ask the patient which treatment option he/she prefers
  - You and the patient thoroughly weigh the different treatment options
  - You and the patient select a treatment option together
  - You and the patient reach an agreement on how to proceed
  - Other, namely:
  - None of the above

*YOUR OPINION ON THE CURRENT TREATMENT DECISION-MAKING PROCESS*

*Please read the statements below and indicate to what extent you agree with them. Think back* ***at recent consultations*** *where a decision (e.g., decision to stop or change therapy, decision to go for a transplant, decision to go into a clinical trial) had to be made concerning treatment (the decision to discontinue or not start a treatment can also be seen as a treatment option) for a myeloma patient.*

- I made clear to my patient that a decision needs to be made
  - Completely disagree
  - Strongly disagree
  - Somewhat disagree
  - Somewhat agree
  - Strongly agree
  - Completely agree
  - N/A
- I wanted to know exactly from my patient how he/she wants to be involved in making the decision
  - Completely disagree
  - Strongly disagree
  - Somewhat disagree
  - Somewhat agree
  - Strongly agree
  - Completely agree
  - N/A
- I told my patient that there are different options for treating his/her medical condition
  - Completely disagree
  - Strongly disagree
  - Somewhat disagree
  - Somewhat agree
  - Strongly agree
  - Completely agree
  - N/A

- I precisely explained the advantages and disadvantages of the treatment options to my patient
  - Completely disagree
  - Strongly disagree
  - Somewhat disagree
  - Somewhat agree
  - Strongly agree
  - Completely agree
  - N/A
- I helped my patient understand all the information
  - Completely disagree
  - Strongly disagree
  - Somewhat disagree
  - Somewhat agree
  - Strongly agree
  - Completely agree
  - N/A
- I asked my patient which treatment option he/she prefers
  - Completely disagree
  - Strongly disagree
  - Somewhat disagree
  - Somewhat agree
  - Strongly agree
  - Completely agree
  - N/A
- My patient and I thoroughly weighed the different treatment options
  - Completely disagree
  - Strongly disagree
  - Somewhat disagree
  - Somewhat agree
  - Strongly agree
  - Completely agree
  - N/A
- My patient and I selected a treatment option together
  - Completely disagree
  - Strongly disagree
  - Somewhat disagree
  - Somewhat agree
  - Strongly agree
  - Completely agree
  - N/A
- My patient and I reached an agreement on how to proceed
  - Completely disagree
  - Strongly disagree
  - Somewhat disagree
  - Somewhat agree
  - Strongly agree
  - Completely agree
  - N/A

*YOUR OPINION ON THE IDEAL LEVEL OF INVOLVEMENT IN TREATMENT DECISION-MAKING*

*To complete the following questions, please consider* ***recent consultations*** *where a decision had to be made concerning treatment.*

- How frequently do you assess the patient’s preferred level of involvement in the decision-making process?
  - Always
  - Very often
  - Sometimes
  - Rarely
  - Never
- IF ANSWERED always, very often, sometimes, rarely: How do you assess the patient’s preferred level of involvement?
- To what extent do **you involve patients** in the decision-making processes **during the initial consultations** **following diagnosis**?
  - Extremely
  - Quite a bit
  - Somewhat
  - A little bit
  - Not at all
- To what extent do **you involve patients** in the decision-making processes **when they are in a later stage of their disease?**
  - Extremely
  - Quite a bit
  - Somewhat
  - A little bit
  - Not at all
- To what extent do **you involve patients** in the decision-making process **regarding whether they should have a transplant?**
  - Extremely
  - Quite a bit
  - Somewhat
  - A little bit
  - Not at all

8. **SOURCES OF INFORMATION**

*YOUR OPINION ON THE INFORMATION SHARING IN TREATMENT DECISION-MAKING*

- How frequently do you assess how patients would like to receive information concerning their disease and treatment options?
  - Always
  - Very often
  - Sometimes
  - Rarely
  - Never
- IF ANSWERED always, very often, sometimes, rarely: How do you provide information to patients concerning their disease and treatment options? (Multiple options possible)
  - Oral discussion
  - Referring to patient organizations, patient support groups
  - Providing information leaflets
  - Providing videos
  - Referring to social media (e.g., YouTube, Instagram, Facebook)
  - Referring to websites
  - Other, please specify:
- Who created the **information leaflet(s)** you provide to patients? (Multiple options possible)
  - Patient organizations, patient support groups
  - Healthcare professionals
  - Pharmaceutical companies
  - Healthcare authorities (such as the European Medicines Agency)
  - Other, please specify:
- Who created the **video(s)** you provide to patients? (Multiple options possible)
  - Patient organizations, patient support groups
  - Healthcare professionals
  - Pharmaceutical companies
  - Healthcare authorities (such as the European Medicines Agency)
  - Other, please specify:
- Who created the **website(s)** you provide to patients? (Multiple options possible)
  - Patient organizations, patient support groups
  - Healthcare professionals
  - Pharmaceutical companies
  - Healthcare authorities (such as the European Medicines Agency)
  - Other, please specify:

9. **IDEAL ROLE FOR PATIENTS AND HEALTHCARE PROFESSIONALS**
   - How would you like a decision between treatment options to be made?
     - I want to make the decision **myself**, **without considering** the opinion of **my patient**
     - I want to make the decision **together with the multidisciplinary team**, **without considering** the opinion of **my patient**
     - I want to make the decision **myself**, **considering** the opinion of **my patient**
     - I want to make the decision **together** with the **multidisciplinary team**, **considering** the opinion of **my patient**
     - I want to make the decision **together with my patient**
     - I want to make the decision **together with the multidisciplinary team** and **the patient**
     - I want **my patient** to make the decision **himself/herself**, **considering my opinion**
     - I want **my patient** to make the decision **himself/herself**, **considering my opinion** and the one of the **multidisciplinary team**
     - I want **my patient** to make the decision **himself/herself**, **without considering my opinion**
     - I want **my patient** to make the decision **himself/herself**, **without considering my opinion** and the one of the **multidisciplinary team**
     - Other, please specify:
   - How would you like a decision between treatment options to be made?
     - I want the **treating physician** to make the decision, **without considering** the opinion of **the patient**
     - I want the **treating physician** to make the decision **together with the multidisciplinary team, without considering** the opinion of **the patient**
     - I want **the treating physician** to make the decision, **considering** the opinion of **the patient**
     - I want the **treating physician** to make the decision **together with the multidisciplinary team, considering** the opinion of **the patient**
     - I want the **treating physician** to make the decision **together with the patient**
     - I want the **treating physician** to make the decision **together** with a **multidisciplinary team** and **the patient**
     - I want **the patient** to make the decision **himself/herself,** **considering** the opinion of **the treating physician**
     - I want **the patient** to make the decision **himself/herself**, **considering** the opinion of **the treating physician** and the one of **the multidisciplinary team**
     - I want **the patient** to make the decision **himself/herself**, **without considering** the opinion of **the treating physician**
     - I want **the patient** to make the decision **himself/herself**, **without considering** the opinion of **the treating physician** and the one of **the multidisciplinary team**
     - Other, please specify:
   - What do you think is needed for you to achieve your ideal role in (treatment) decision-making processes?
   - Which healthcare professional(s) is/are currently involved in the (treatment) decision-making processes? (Multiple options possible)
     - Haematologist
     - Oncologist
     - Haemato-oncologist
     - Nurse specialist
     - Nurse
     - Other, please specify:
   - Which healthcare professional(s) do you think can have a (more prominent) role than presently in the (treatment) decision-making processes? (Multiple options possible)
     - Haematologist
     - Oncologist
     - Haemato-oncologist
     - Nurse specialist
     - Nurse
     - Other, please specify:
     - None
   - In which steps of the decision-making process do you think **haematologists** can have a (more prominent) role than presently? (Multiple options possible)
     - *Making clear that a decision needs to be made*
     - *Wanting to know exactly how the patient wants to be involved in making the decision*
     - *Telling the patient that there are different options for treating their medical condition*
     - *Precisely explaining the advantages and disadvantages of the treatment options*
     - *Helping the patient understand all the information*
     - *Asking the patient which treatment option he/she prefers*
     - *Thoroughly weighing the different treatment options*
     - *Selecting a treatment option together*
     - *Reaching an agreement on how to proceed*
     - *All of the above*
     - *None of the above*
     - *Other, please specify:*
   - In which steps of the decision-making process do you think **oncologists** can have a (more prominent) role than presently? (Multiple options possible)
     - *Making clear that a decision needs to be made*
     - *Wanting to know exactly how the patient wants to be involved in making the decision*
     - *Telling the patient that there are different options for treating their medical condition*
     - *Precisely explaining the advantages and disadvantages of the treatment options*
     - *Helping the patient understand all the information*
     - *Asking the patient which treatment option he/she prefers*
     - *Thoroughly weighing the different treatment options*
     - *Selecting a treatment option together*
     - *Reaching an agreement on how to proceed*
     - *All of the above*
     - *None of the above*
     - *Other, please specify:*
   - In which steps of the decision-making process do you think **haemato-oncologists** can have a (more prominent) role than presently? (Multiple options possible)
     - *Making clear that a decision needs to be made*
     - *Wanting to know exactly how the patient wants to be involved in making the decision*
     - *Telling the patient that there are different options for treating their medical condition*
     - *Precisely explaining the advantages and disadvantages of the treatment options*
     - *Helping the patient understand all the information*
     - *Asking the patient which treatment option he/she prefers*
     - *Thoroughly weighing the different treatment options*
     - *Selecting a treatment option together*
     - *Reaching an agreement on how to proceed*
     - *All of the above*
     - *None of the above*
     - *Other, please specify:*
   - In which steps of the decision-making process do you think **nurse specialists** can have a (more prominent) role than presently? (Multiple options possible)
     - *Making clear that a decision needs to be made*
     - *Wanting to know exactly how the patient wants to be involved in making the decision*
     - *Telling the patient that there are different options for treating their medical condition*
     - *Precisely explaining the advantages and disadvantages of the treatment options*
     - *Helping the patient understand all the information*
     - *Asking the patient which treatment option he/she prefers*
     - *Thoroughly weighing the different treatment options*
     - *Selecting a treatment option together*
     - *Reaching an agreement on how to proceed*
     - *All of the above*
     - *None of the above*
     - *Other, please specify:*
   - In which steps of the decision-making process do you think **nurses** can have a (more prominent) role than presently? (Multiple options possible)
     - *Making clear that a decision needs to be made*
     - *Wanting to know exactly how the patient wants to be involved in making the decision*
     - *Telling the patient that there are different options for treating their medical condition*
     - *Precisely explaining the advantages and disadvantages of the treatment options*
     - *Helping the patient understand all the information*
     - *Asking the patient which treatment option he/she prefers*
     - *Thoroughly weighing the different treatment options*
     - *Selecting a treatment option together*
     - *Reaching an agreement on how to proceed*
     - *All of the above*
     - *None of the above*
     - *Other, please specify:*
   - In which steps of the decision-making process do you think **the other people you indicated in the previous question** can have a (more prominent) role than presently? (Multiple options possible)
     - *Making clear that a decision needs to be made*
     - *Wanting to know exactly how the patient wants to be involved in making the decision*
     - *Telling the patient that there are different options for treating their medical condition*
     - *Precisely explaining the advantages and disadvantages of the treatment options*
     - *Helping the patient understand all the information*
     - *Asking the patient which treatment option he/she prefers*
     - *Thoroughly weighing the different treatment options*
     - *Selecting a treatment option together*
     - *Reaching an agreement on how to proceed*
     - *All of the above*
     - *None of the above*
     - *Other, please specify:*

*FEEDBACK QUESTIONS:*

- What did you think about the length of the survey?
  - Way too long
  - Too long
  - Manageable
  - Just right
- How easy or difficult was it for you to understand the questions?
  - Very easy
  - Easy
  - Difficult
  - Very difficult
- How easy or difficult was it for you to answer the questions?
  - Very easy
  - Easy
  - Difficult
  - Very difficult
- Do you have any suggestions for improving the survey? If so, please describe them here. If you do not have any remarks, please enter a "/" or type "NA".
