# Supplementary material for: Enhancing patient involvement through shared decision-making: Are we there yet in multiple myeloma? Findings from a cross-country survey
Source: PLoS One. 2026 Aug 28;21(8):e0356989. doi: 10.1371/journal.pone.0356989 (PMC13524276; doi:10.1371/journal.pone.0356989)
Supplement: Supplementary file 2 [file pone.0356989.s002.docx]

S2. Appendix 2 – Knowledge of the ‘shared decision-making (SDM) concept

Figure 1 Description SDM concept

Associations participant characteristics and having heard of the SDM concept

Patients

Table 1 Associations patients characteristics and having heard of the SDM concept. *Since there are very few events for gender other than ‘male’ and ‘female’ only these two categories are kept for analysis.

| **Association tested** | **Significant?** | **Test** | **P-value** |
| --- | --- | --- | --- |
| Age and having heard of the SDM concept | no | Wilcoxon rank sum test | 0.10 |
| Health literacy and having heard of the SDM concept | no | Wilcoxon rank sum test | 0.6 |
| Gender and having heard of the SDM concept | yes | Fisher’s test p-value (simulated)* | 0.007 |
| Country and having heard of the SDM concept | yes | Fisher’s test p-value (simulated) | <0.001 |
| Level of education and having heard of the SDM concept | yes | Fisher’s test p-value (simulated) | 0.009 |
| Health status and having heard of the SDM concept | yes | Wilcoxon rank sum test | 0.004 |
| Treatment location and having heard of the SDM concept | no | Fisher’s test p-value (simulated) | 0.8 |
| Treatment duration and having heard of the SDM concept | no | Wilcoxon rank sum test | 0.7 |
| Treatment lines and having heard of the SDM concept | no | Fisher’s test p-value (simulated) | 0.076 |
| Medical background and having heard  of the SDM concept | yes | Fisher’s test p-value (simulated) | 0.001 |
| Native language spoken in the hospital and having heard of the SDM concept | no | Fisher’s test p-value (simulated) | >0.9 |

Table 2 Patients – having heard of the SDM concept with gender

| Gender | Having heard of the SDM concept | |
| --- | --- | --- |
|  | Yes | No |
| Male | 108 | 178 |
| Female | 133 | 136 |
| Non-binary/gender non-conforming | 1 | 0 |
| Other, please specify | 0 | 1 |
| Prefer not to say | 0 | 1 |
| Fisher's test p-value (simulated) on Male and Female only: 0.007 | | |

Table 3 Patients – having heard of the SDM concept with country

| Country | Having heard of the SDM concept | |
| --- | --- | --- |
|  | Yes | No |
| Italy | 1 | 0 |
| Portugal | 6 | 3 |
| UK | 4 | 2 |
| Brazil | 1 | 0 |
| The Netherlands | 57 | 103 |
| Czech Republic | 1 | 0 |
| Belgium | 30 | 58 |
| Finland | 1 | 2 |
| England | 1 | 0 |
| Norway | 1 | 0 |
| France | 3 | 0 |
| Switzerland | 4 | 5 |
| Israel | 80 | 51 |
| US | 1 | 0 |
| Slovenia | 10 | 18 |
| Spain | 5 | 7 |
| Sweden | 21 | 28 |
| Germany | 15 | 34 |
| Ireland | 0 | 2 |
| Hungary | 0 | 1 |
| Romania | 0 | 1 |
| Macedonia | 0 | 1 |
| Fisher's test p-value (simulated): <0.001 | | |

Table 4 Patients – having heard of the SDM concept with level of education

| Level of education | Having heard of the SDM concept | |
| --- | --- | --- |
|  | Yes | No |
| I did not finish high school | 6 | 10 |
| High school | 55 | 105 |
| Bachelor's degree | 62 | 93 |
| Master’s degree | 65 | 53 |
| Doctorate | 21 | 14 |
| Other, please specify: | 33 | 41 |
| Fisher's test p-value (simulated): 0.009 | | |

Table 5 Patients – having heard of the SDM concept with health status

| Having heard of the SDM concept | N | Health status |
| --- | --- | --- |
| Yes | 242 | 45.02 (34 - 55) |
| No | 316 | 40.97 (31.75 - 53) |
| Statistic: mean (Q1 - Q3) | | |
| Wilcoxon rank sum test p-value: 0.004 | | |

Table 6 Patients – having heard of the SDM concept with medical background

| Medical background | Having heard of the SDM concept | |
| --- | --- | --- |
|  | Yes | No |
| Yes | 47 | 29 |
| No | 190 | 282 |
| I am not sure | 5 | 5 |
| Fisher’s test p-value (simulated): 0.001 | | |

Healthcare professionals

Table 7 Associations HCPs characteristics and having heard of the SDM concept. The p-value for country could not be calculated given the very low count of HCPs in each county category. *2 gender categories, male and female.

| **Association tested** | **Significant?** | **Test** | **P-value** |
| --- | --- | --- | --- |
| Age and having heard of the SDM concept | no | Wilcoxon rank sum test | 0.066 |
| Gender and having heard of the SDM concept | no | Pearson's Chi-squared test* | 0.2 |
| HCP type and having heard of the SDM concept | no | Fisher's Exact Test for Count Data with simulated p-value | 0.6 |
| Workplace and having heard of the SDM concept | no | Fisher's Exact Test for Count Data with simulated p-value | 0.5 |
| Years of experience and having heard of the SDM concept | no | Wilcoxon rank sum test | 0.2 |
| Minutes per consultation and having heard of the SDM concept | no | Wilcoxon rank sum test | 0.5 |
| Currently involved in research and having heard of the SDM concept | no | Pearson's Chi-squared test | 0.2 |
| Currently involved in guideline development and having heard of the SDM concept | no | Pearson's Chi-squared test | 0.6 |
