# Supplementary material for: Enhancing patient involvement through shared decision-making: Are we there yet in multiple myeloma? Findings from a cross-country survey
Source: PLoS One. 2026 Aug 28;21(8):e0356989. doi: 10.1371/journal.pone.0356989 (PMC13524276; doi:10.1371/journal.pone.0356989)
Supplement: Supplementary file 3 [file pone.0356989.s003.docx]

S3. Appendix 3 – Current patient involvement

Involvement during consultations

Table 1 median SDM score HCPs and patients; P-value for t-test between the HCP and patient groups for the SDMQ-9

| **Characteristic** | **HCP**  N = 89^1^ | **Patient**  N = 558^1^ | **p-value**^2^ |
| --- | --- | --- | --- |
| sdmq9 | 78 (71, 84) | 58 (42, 71) | <0.001 |
| ^1^Median (Q1, Q3) | | | |
| ^2^Welch Two Sample t-test | | | |

Table 2 Relationship with the treating physician against involvement in recent consultations

| ***Relationship with physician*** | ***Involvement in recent consultations*** | | | | | | |
| --- | --- | --- | --- | --- | --- | --- | --- |
|  | *Extremely* | *Quite a bit* | *Somewhat* | *A little bit* | *Not at all* | *I do not know* | *n (%)* |
| *Very good* | 85 | 128 | 54 | 28 | 16 | 6 | 317 (56.81) |
| *Good* | 21 | 67 | 35 | 32 | 14 | 4 | 173 (31.00) |
| *Neither bad nor good* | 2 | 9 | 9 | 20 | 19 | 2 | 61 (10.93) |
| *Bad* | 0 | 1 | 2 | 0 | 1 | 0 | 4 (0.72) |
| *Very bad* | 0 | 0 | 0 | 2 | 1 | 0 | 3 (0.54) |
| *n (%)* | 108 (19.4) | 205 (36.2) | 100 (17.9) | 82 (14.7) | 51 (9.1) | 12 (2.2) | 558 (100) |

Table 3 Mean SDMQ-9 score for each category of relationship with treating physician

| **Relationship with physician** | N | **SDMQ-9** |
| --- | --- | --- |
| Very bad | 3 | 20 (20 - 20) |
| Bad | 4 | 36.3 (17.78 - 46.67) |
| Neither bad nor good | 61 | 39.21 (23.89 - 50) |
| Good | 173 | 45.45 (32.78 - 60) |
| Very good | 317 | 60.51 (49.44 - 75.56) |
| Statistic: mean (Q1 - Q3) | | |
| ANOVA p-value: <0.001 | | |

*Table 4 Relationship with the treating physician against asked the patient the preferred way of receiving information*

| **Relationship with physician** | **Preferred way of receiving information assessed** | | | |
| --- | --- | --- | --- | --- |
|  | Yes | No | I am not sure | n (%) |
| Very good | 68 | 204 | 45 | 317 (56.81) |
| Good | 17 | 131 | 25 | 173 (31.00) |
| Neither bad nor good | 1 | 56 | 4 | 61 (10.93) |
| Bad | 0 | 4 | 0 | 4 (0.72) |
| Very bad | 0 | 3 | 0 | 3 (0.54) |
| n (%) | 86 (15.41) | 398 (71.33) | 74 (13.26) | 558 (100) |

Figure 1 Involvement of patients at recent consultations and at diagnosis
