# Supplementary material for: Enhancing patient involvement through shared decision-making: Are we there yet in multiple myeloma? Findings from a cross-country survey
Source: PLoS One. 2026 Aug 28;21(8):e0356989. doi: 10.1371/journal.pone.0356989 (PMC13524276; doi:10.1371/journal.pone.0356989)
Supplement: Supplementary file 4 [file pone.0356989.s004.docx]

S4. Appendix 4 – Desired patient involvement

Reasons and barriers of involvement

Table 1 Associations patient characteristics and patient involvement. *Since there are very few events for gender other than ‘male’ and ‘female’ only these two categories are kept for analysis.

| **Association tested** | **Significant?** | **Test** | **P-value** |
| --- | --- | --- | --- |
| Gender and patient involvement | No | Fisher’s test p-value (simulated)* | >0.9 |
| Age and patient involvement | No | Wilcoxon rank sum test | 0.4 |
| Country and patient involvement | Yes | Fisher’s test p-value (simulated) | 0.033 |
| Health literacy and patient involvement | No | Wilcoxon rank sum test | 0.8 |
| Level of education and patient involvement | No | Fisher’s test p-value (simulated) | 0.2 |
| Health status and patient involvement | No | Wilcoxon rank sum test p-value | >0.9 |
| Treatment location and patient involvement | No | Fisher’s test p-value (simulated) | 0.7 |
| Treatment duration and patient involvement | No | Wilcoxon rank sum test | >0.9 |
| Treatment lines and patient involvement | No | Fisher’s test p-value (simulated) | 0.064 |
| Medical background and patient involvement | No | Fisher’s test p-value (simulated) | 0.13 |
| Native language spoken in the hospital and patient involvement | No | Fisher’s test p-value (simulated) | >0.9 |

Table 2 Patients - involvement with country. Since the count table is larger than 2 x 2 table, Monte Carlo simulation is used to obtain a p-value.

| Country | Involvement | |
| --- | --- | --- |
|  | Yes | No |
| Italy | 1 | 0 |
| Portugal | 9 | 0 |
| UK | 6 | 0 |
| Brazil | 1 | 0 |
| The Netherlands | 159 | 1 |
| Czech Republic | 1 | 0 |
| Belgium | 84 | 4 |
| Ireland | 2 | 0 |
| Finland | 3 | 0 |
| England | 1 | 0 |
| Hungary | 1 | 0 |
| Norway | 1 | 0 |
| France | 3 | 0 |
| Switzerland | 9 | 0 |
| Israel | 121 | 10 |
| US | 1 | 0 |
| Slovenia | 26 | 2 |
| Macedonia | 1 | 0 |
| Spain | 10 | 2 |
| Sweden | 47 | 2 |
| Germany | 49 | 0 |
| Romania | 0 | 1 |
| Fisher's test p-value (simulated): 0.033 | | |

Desired level of involvement during consultations

Table 3 Correlation between current involvement and desired involvement. Correlations are analysed using a Spearman correlation.

| Variable 1 | Variable 2 | Correlation | P-value |
| --- | --- | --- | --- |
| Current involvement **(diagnosis)** | Desired involvement **(diagnosis)** | 0.44 | <0.001 |
| Current involvement **(diagnosis)** | Current involvement **(recent)** | 0.66 | <0.001 |
| Desired involvement **(recent)** | Desired involvement **(diagnosis)** | 0.76 | <0.001 |
| Desired involvement **(recent)** | Current involvement **(recent)** | 0.51 | <0.001 |

The graphs show the distribution of answers patients gave on the questions on involvement. Patients on the diagonals in the graphs answered the same on the questions on x and y axes (Figure 1).

Figure 1 Current and desired involvement at diagnosis and at recent consultations
